# DNA barcoding the flowering plants from the tropical coral islands of Xisha (China)

**DOI:** 10.1002/ece3.4545

**Published:** 2018-10-03

**Authors:** Shengchun Li, Xin Qian, Zexin Zheng, Miaomiao Shi, Xiaoyu Chang, Xiaojuan Li, Junfang Liu, Tieyao Tu, Dianxiang Zhang

**Affiliations:** ^1^ Key Laboratory of Plant Resources Conservation and Sustainable Utilization, South China Botanical Garden Chinese Academy of Sciences Guangzhou China; ^2^ University of Chinese Academy of Sciences Beijing China; ^3^ South China Agricultural University Guangzhou China

**Keywords:** biodiversity conservation, community assembly, DNA barcode, Pacific Ocean, South China Sea, species identification

## Abstract

**Aim:**

DNA barcoding has been widely applied to species diversity assessment in various ecosystems, including temperate forests, subtropical forests, and tropical rain forests. However, tropical coral islands have never been barcoded before due to the difficulties in field exploring. This study aims at barcoding the flowering plants from a unique ecosystem of the tropical coral islands in the Pacific Ocean and supplying valuable evolutionary information for better understanding plant community assembly of those particular islands in the future.

**Location:**

Xisha Islands, China.

**Methods:**

This study built a DNA barcode database for 155 plant species from the Xisha Islands using three DNA markers (ITS, *rbc*L, and *mat*K). We applied the sequence similarity method and a phylogenetic‐based method to assess the barcoding resolution.

**Results:**

All the three DNA barcodes showed high levels of PCR success (96%–99%) and sequencing success (98%–100%). ITS performed the highest rate of species resolution (>95%) among the three markers, while plastid markers delivered a relatively poor species resolution (85%–90%). Our analyses obtained a marginal increase in species resolution when combining the three DNA barcodes.

**Main conclusions:**

This study provides the first plant DNA barcode data for the unique ecosystem of tropical coral islands and considerably supplements the DNA barcode library for the flowering plants on the oceanic islands. Based on the PCR and sequencing success rates, and the discriminatory power of the three DNA regions, we recommend ITS as the most successful DNA barcode to identify the flowering plants from Xisha Islands. Due to its high sequence variation and low fungal contamination, ITS could be a preferable candidate of DNA barcode for plants from other tropical coral islands as well. Our results also shed lights on the importance of biodiversity conservation of tropical coral islands.

## 1 INTRODUCTION

The primary application of DNA barcoding is to identify unknown samples, and the emergence of DNA barcoding has greatly promoted the survey of biodiversity (Gregory, 2005). DNA barcoding has been particularly valuable in the inventorying of biodiversity hotspots. Successful investigations have been carried out in Mount Kinabalu, Malaysia (Merckx et al., 2015), and Ontario, Canada (Telfer et al., 2015), providing a convenient and efficient way for recognition of nature in these regions. DNA barcoding can also be a powerful tool for addressing fundamental questions in ecology, evolution, and conservation biology (Kress, García‐Robledo, Uriarte, & Erickson, 2015). A considerable number of cryptic and new species have been discovered based on evidence from DNA barcodes (García‐Robledo, Kuprewicz, Staines, Kress, & Erwin, 2013; Hamsher & Saunders, 2014; Hebert, Penton, Burns, Janzen, & Hallwachs, 2004; Silva, de Abreu, Orlando, Wisniewski, & Santos‐Wisniewski, 2014; Smith et al., 2012; Winterbottom, Hanner, Burridge, & Zur, 2014). DNA barcoding data prodigiously contribute to understanding the evolutionary relations within a given community (Kress, et al., 2009). As molecular information makes the interpretation of phylogenetic relationships easier and more reliable (Webb, Ackerly, McPeek, & Donoghue, 2002), DNA barcoding data could also be useful in plant community ecology concerning the relationships among coexisting species. Since its recent consummation, the DNA barcoding library has become a standard metric for biological conservation assessment (Faith, 1992, 2008 ; Forest, et al., 2007). DNA sequence data provide an evolutionary dimension to diversity estimates, which is of great value to compare diversity and establish protected areas across the landscape (Kress et al., 2015).

The development of sequencing technologies and their broad usage in species identification, biodiversity assessment, and ecological researches have greatly promoted the studies in DNA barcoding. During the last 15 years, scientists have published more than 3,000 papers on plant DNA barcoding–covering different vegetation types in different climate zones, including arctic flora of Canada (Kuzmina, Johnson, Barron, & Hebert, 2012; Saarela, Sokoloff, Gillespie, Consaul, & Bull, 2013), northern temperate forests (Burgess et al., 2011; Fazekas et al., 2008), subtropical forests of China (Liu et al., 2015), and tropical forests in the Old World as well as the New World (Costion et al., 2011; Kress et al., 2009; Parmentier et al., 2013).

Tropical coral islands represent a unique ecosystem: (a) They are far away from continental ecological systems with clear oceanic boundaries; (b) Their species composition could be very different from those of the mainland; (c) They often represent small geographical areas, where the species pool may come either from closely related species or from distantly related clades; (d) They are of particular conservation and scientific interests in the global inventory of biodiversity (Monaghan, Balke, Pons, & Vogler, 2006), and they badly need a comprehensive understanding on biodiversity and ecology due to the increasingly anthropogenic disturbance.

The flora of tropical oceanic islands has rarely been studied so far. The extreme difficulties of plant exploring and sampling from these areas might be the major limiting factors. The high cost of exploring these islands, combined with unstable weather condition, also contributes to the poor knowledge concerning their plant biodiversity. Although both the Chinese version Flora Reipublicae Popularis Sinicae (FRPS) (1959–2004) and the English version Flora of China were fully compiled (Wu, Raven, & Hong, 1994–2013), the collections of plant specimens from the oceanic islands are still rare. Of the ca. 1 million plant specimens in the herbarium of South China Botanical Garden (IBSC), which hosts specimens mainly from southern China, only 2,643 collections are from the ca. 6,500 oceanic islands of China (excluding Hainan Island and Taiwan Island) from 1927 to 2013.

**Figure 1:**
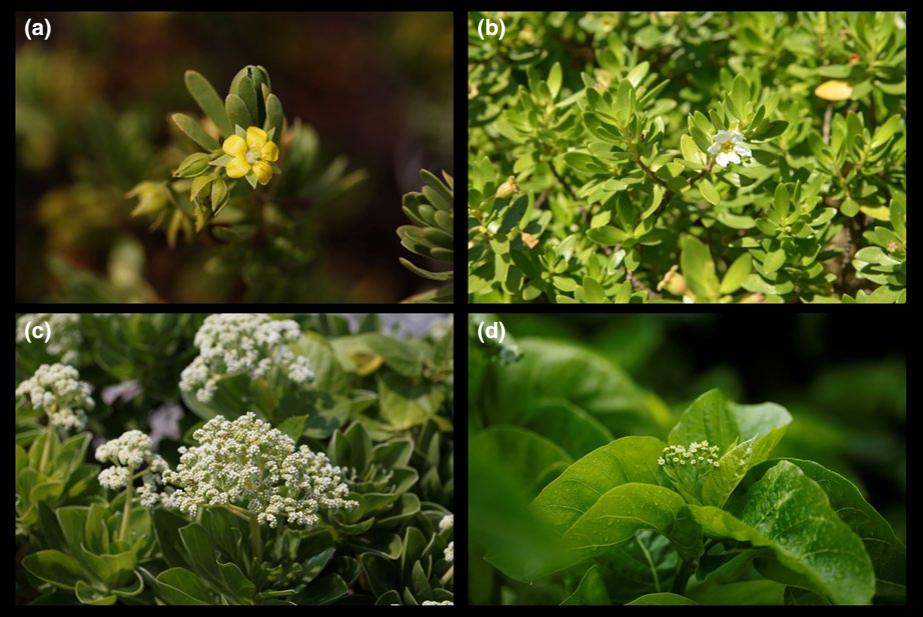
Typical plants on Xisha Islands. (a) *Suriana maritima* L.; (b) *Pemphis acidula* J. R. Forst. & G. Forst.; (c) *Tournefortia argentea* L. f.; (d) *Pisonia grandis* R. Br.

Xisha Islands are a group of tropical oceanic coral islands with ca. 25 small islets in the South China Sea and are about 330 km apart from Hainan Island. Xisha Islands with a total land area of ca. 7.6 km^2^ are very distinctive from other ecosystems in China, including mainland China, as well as the many continental islands and the oceanic volcanic islands. The soil of Xisha Islands is basically made of phospho calcic soil and coastal saline soil that developed from coral and shell remnants, and is often dominant with salt‐tolerant plant species. Plants on these islands may also be highly constrained by extreme physical components of the environment, such as high temperature, strong solar radiation, and severe wind and drought stress. As a result, Xisha Islands harbor a number of plant species that are rarely recorded in mainland China or on the many continental islands, especially the dominant species such as *Suriana maritima* L. (Surianaceae), *Pemphis acidula* J.R. Forst. & G. Forst. (Lythraceae), *Guettarda speciosa* L. (Rubiaceae), *Scaevola taccada* (Gaertn.) Roxb. (Goodeniaceae), *Pisonia grandis* R. Br. (Nyctaginaceae), and *Tournefortia argentea* L. f. (Boraginaceae) (Tong, Jian, Chen, Li, & Xing, 2013) (Figure 1).

Since 2014, we have explored the flora of 19 islands of the Xisha Islands and conducted a comprehensive plant sampling by species through different seasons. Using the technology of DNA barcoding, we would like to provide a particular case study of plant DNA barcoding in this unique flora. Totally, we tested three DNA regions (ITS, *rbc*L, and *mat*K) for 155 species in 120 genera of 42 families representing nearly all native plant species from Xisha Islands. Phylogenetic relations among species can help reconstruct evolutionary relationships within communities and allow people to infer how the biodiversity has come about (Pagel, 1999). Unfortunately, the full diversity of species in most regions is still unknown and information on the DNA sequences is extremely poor, so that understanding the mechanisms of maintaining biodiversity patterns has remained elusive (Purvis & Hector, 2000). Our study filled the gap on the barcode library from tropical coral islands in the Pacific Ocean and recovered the lineage relationships for this unique flora. Our study provides a precise snapshot of plant biodiversity in tropical oceanic coral islands, and the DNA sequence data could contribute to comprehensively understanding biodiversity patterns of island floras in future studies.

## 2 METHODS

### 2.1 Taxon sampling and data collection

We conducted our field work for the DNA samples on the Xisha Islands from 2014 to 2017. We sampled 312 individuals representing 155 species in 120 genera of 42 angiosperm families, of which 132 species have been sampled at least twice. Considering that there is little intraspecific variation due to the similar geography and climate conditions, we only sampled two individuals of most species. We sampled individuals of the same species from different islands as far as possible to guarantee that the two individuals represent distinct lineages. We also explored the islands at both dry and wet seasons to make sure that the samples bear flowers and fruits, and thus can be correctly identified based on morphology. All the voucher specimens were deposited at Herbarium of the South China Botanical Garden (IBSC). A list of the plant samples and the GenBank accessions are provided in Supporting Information Table S1.

### 2.2 DNA extraction, PCR, and sequencing

Total plant DNA was extracted from dried leaves preserved through silica gel desiccation using the CTAB method (Doyle & Doyle, 1987). We selected two plastid DNA regions *rbc*L, *mat*K, and the nuclear ribosomal internal transcribed spacer (ITS) for routine PCR. The PCR amplification for plastid DNA regions used an initial predenaturation step at 94°C for 5 min, followed by 35 cycles of denaturing for 1 min at 94°C, primer annealing for 30 s at 52°C, and elongation step for 1 min at 72°C, with a final 8 min elongation step at 72°C. The PCR cycling condition of ITS was the same as the plastid DNA, except for primer annealing at 53°C. All samples were sequenced directly in both directions using the same PCR primers. Details on the primer pairs used for amplifying each locus are provided in Table 1.

**Table 1:** Primer pairs for three DNA barcode regions: *rbc*L, *mat*K, and ITS

Marker	Primer	Sequence(5′−3″)	References
*rbc*L	1F	ATGTCACCACAAACAGAAAC	Fay, Swensen, and Chase (1997)
	1379R	GCAGCTAATTCAGGACTCC	Little and Barrington, (2003)
*mat*K	KIM 3F	CGTACAGTACTTTTGTGTTTACGAG	K. J. Kim, unpublished
	KIM 1R	ACCCAGTCCATCTGGAAATCTTGGTTC	K. J. Kim, unpublished
	390F	CGATCTATTCATTCAATATTTC	Cuénoud et al. (2002)
	1326R	TCTAGCACACGAAAGTCGAAGT	Cuénoud et al. (2002)
	Kew xF	TAATTTACGATCAATTCATTC	Ford, Ayres, Toomey, and Liu (2009)
	Kew 5R	GTTCTAGCACAAGAAAGTCG	Ford et al. (2009)
ITS	*N*‐nc18s10 F	AGGAGAAGTCGTAACAAG	Suh, Thien, Reeve, and Zimmer (1996), Wen and Zimmer, (1996)
	C26A R	GTTTCTTTTCCTCCGCT	Suh et al. (1996)

### 2.3 Data analyses

Sequences were assembled using SEQUENCHER 5.3 (Gene Codes Corporation 2015) and aligned with Geneious 8.1.7 (https://www.geneious.com, Kearse et al., 2012). The plastid DNA markers *rbc*L and *mat*K were easily aligned using MAFFT alignment in Geneious. The ITS sequences were first aligned automatically via Geneious for all samples, then we retained three parts of conservative sequences for all samples, the remaining parts of variant sequences were partitioned by families. We applied two analytical methods to assess the barcoding resolution, the sequence similarity method (BLASTn searches) and a phylogenetic‐based method. BLASTn method (Altschul, Gish, Miller, Myers, & Lipman, 1990) was operated through BLAST+2.6.0 in which the sequence was correctly determined when that species has the highest Bit‐Score among all candidates.

Phylogenetic tree based on maximum parsimony (MP) algorithms was reconstructed in PAUP* 4.0b10 (Swofford, 2000). We reconstructed phylogenies both for the three markers independently and for their combinations. The supports of nodes were assessed on the basis of 500 bootstrap replicates. Species were considered resolved if a group of samples representing a species were recovered as monophyly with a bootstrap value that exceeded 70% (Theodoridis et al., 2012).

## 3 RESULTS

### 3.1 PCR and sequencing success rates

The PCR and the sequencing success rates were generally high for the three regions. *rbc*L exhibited the highest amplification success rate (99%), followed by *mat*K (97%) and ITS (96%). Similarly, *rbc*L could be successfully sequenced for all individuals, while *mat*K and ITS were for 99% and 98%, respectively. In total, we obtained 902 sequences from 312 individuals of 155 species, including 309 individuals of 154 species for *rbc*L, 299 individuals of 152 species for *mat*K, 294 individuals of 148 species for ITS (Table 2).

**Table 2:** PCR and sequencing success of different DNA barcode regions for 155 flowering plants on Xisha Islands

Marker	PCR success (%)	Sequencing success (%)	Sequences (species)
*rbc*L	99	100	309 (154)
*mat*K	97	98	299 (152)
ITS	96	98	294 (148)

### 3.2 Species discrimination

**Figure 2:**
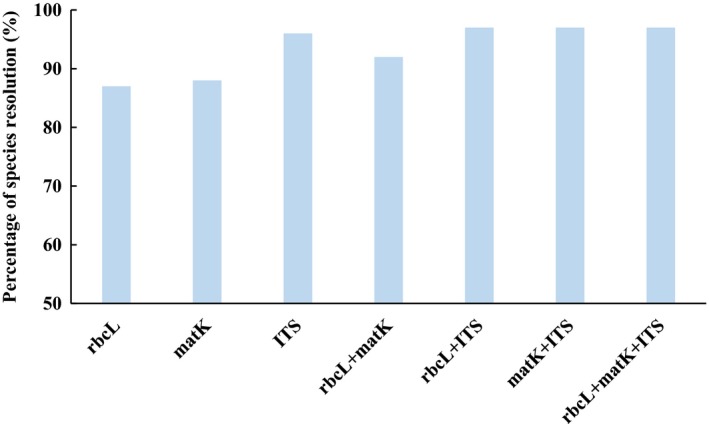
Species resolution for three DNA barcode regions and their combinations using phylogenetic‐based method

Our results showed that the two analytical methods performed similar trends among the three barcodes. BLASTn analysis provided the highest success rate of species‐level resolution with ITS (95%), followed by *mat*K (87%), and then *rbc*L (85%) (Table 3). All markers delivered 100% correct assignments to genus and family. We assessed the discriminatory power of each DNA barcode by evaluating the percentage monophyly of each species using MP trees (Figure 2). Of the single marker analyses, all the three DNA barcodes showed high rates of species resolution. ITS was the most successful at the species‐level resolution (96%), while *rbc*L and *mat*K resolved 87% and 89% of species, respectively. In addition, the combination of *rbc*L + ITS (97%) and *mat*K + ITS (97%) resulted in the highest rate of species resolution, followed by *rbc*L + matK (92%). The three‐barcode combination (*rbc*L + *mat*K + ITS) (97%) did not improve species resolution compared to the two‐barcode combination of ITS with either *rbc*L or *mat*K. The families Poaceae, Fabaceae, Asteraceae, Euphorbiaceae, and Malvaceae each had more than 10 species. The genus *Sida* L. of Malvaceae had seven species, and the genera *Ipomoea* L. (Convolvulaceae), *Euphorbia* L. (Euphorbiaceae), and *Digitaria* Hall. (Poaceae) each had five species. ITS showed higher rates of species identification in these large families and genera than *rbcL* or *matK* except in *Digitaria*. The three DNA barcodes alone and their combination all showed poor species resolution in *Digitaria*.

**Table 3:** BLAST results of species resolution for the three DNA barcode regions

Taxonomic rank	Frequency of correct identification (BLAST)%		
	*rbc*L	*mat*K	ITS
Species	84	86	95
Genus	100	100	100

## 4 DISCUSSION

Primer universality and species identification are two crucial criterions for an ideal DNA barcode. The three DNA barcodes showed high rates of amplification and sequencing successes, among which *rbc*L had the best performance of universality. For *mat*K, three pairs of primers were used due to the lack of universal primers for the broad plant taxa (Costion et al., 2011; Kress, Wurdack, Zimmer, Weigt, & Janzen, 2005; Parmentier et al., 2013). Additionally, we failed to obtain 13 *mat*K sequences, of which 46% belongs to Poales. ITS showed an excellent universality (PCR success rate: 96%; sequencing success rate: 98%), and the fungal contamination was detected in only seven samples (2.2%) of 312 individuals.

Two analytical methods both showed that the three barcodes alone could identified>85% species and ITS performed the best. In the tree‐based method, the combination of ITS and the plastid DNA barcodes did not get a better species resolution than the single ITS barcode, while the combination of two plastid DNA barcodes got even lower species resolution than ITS. Each of the MP trees had considerably high terminal branch supports. Generally, nuclear DNA (nrDNA) evolves rapidly and possesses more variations that can differentiate closely related species (Kress, et al., 2005; Nieto Feliner & Rosselló, 2007; Sass, Little, Stevenson, & Specht, 2007). Plastid DNA region (such as *rbc*L and *mat*K) is more conservative than nrDNA region (such as ITS). Either *rbc*L or *mat*K can well resolve the taxa at genus level or above but does not work well among closely related species or within species‐rich genera.

**Figure 3:**
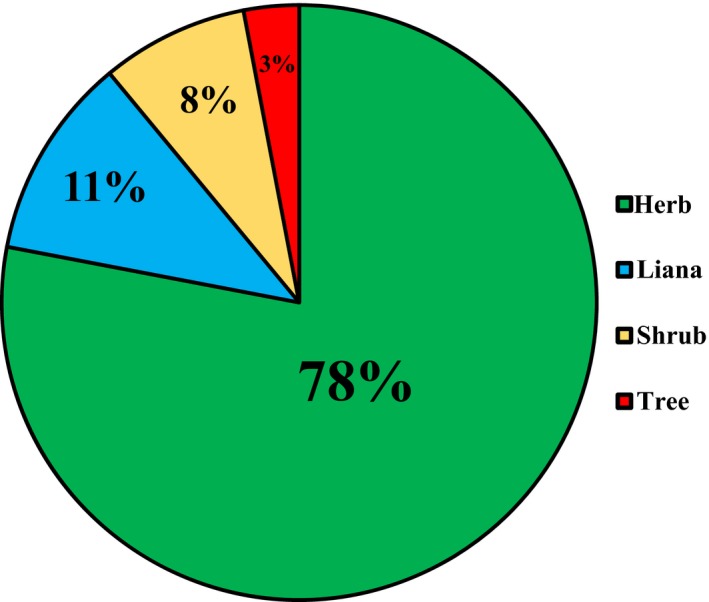
Four types of life forms and their proportion of flowering plants on Xisha Islands

**Figure 4:**
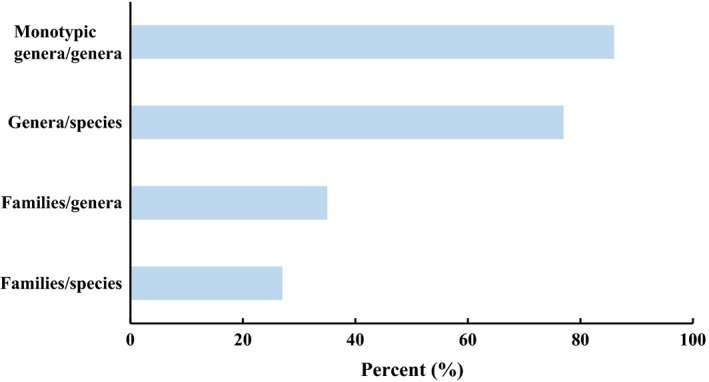
The taxonomic structure of flowering plants on Xisha Islands

To date, thousands of DNA barcoding studies have been carried out on different spatial scales of species pool and various combinations of different loci have been suggested as their preferred barcodes. A number of DNA barcodes from plastid genome have been proposed based on the considerable universality and relatively high discriminatory power (Burgess et al., 2011; CBOL, 2009; Fazekas et al., 2008; Kress & Erickson, 2007; Kress et al., 2009; Parmentier et al., 2013; Pei et al., 2015). In some cases, however, it is especially difficult to distinguish closely related species with plastid DNA barcodes. ITS has been commonly used in plant molecular systematic investigations owing to a high rate of species resolution (Alvarez & Wendel, 2003; Chen et al., 2010; Kress et al., 2005; Li et al., 2011; Yao et al., 2010), but has not been widely applied in DNA barcoding studies because of several limitations: incomplete evolutionary history, fungal contamination, and poor primer universality (Hollingsworth, Graham, & Little, 2011). It could be difficult to be amplified and sequenced from diverse samples because of fungal contamination and divergent paralogue copies caused by incomplete concerted evolution of ITS (Baldwin et al., 1995; Cullings & Vogler, 1998; Hollingsworth et al., 2011; Zhang, Wendel, & Clark, 1997). In this study, our results indicated that ITS could be a better choice for barcoding the flora of the tropical coral islands of Xisha compared to *rbc*L and *mat*K. First, ITS showed outstanding primer universality. The flora of Xisha Islands is mainly composed of herbs (78%) and lianas (11%) (Figure 3). The primer universality of ITS in our study (94%) is much higher than in the study of 285 tropical trees reported by Gonzalez et al. (2009) (41%) and a sample of 531 woody species published by Liu et al. (2015) (73%). The domination by herbs and lianas on Xisha Islands might contribute to the high primer universality. Second, endophytic fungi are considered to have a great effect on plant DNA barcoding when using ITS in different ecosystems (Hollingsworth et al., 2011). However, the fungal contamination could be much lower in the vegetation of the tropical coral islands than in other vegetations. In this study, only seven species (2.2%) were detected with fungal contamination of ITS. Finally, we analyzed the taxonomic structure of plants on Xisha Islands. Interestingly, the genera/species (77%) and monotypic genera/genera (86%) ratios are extraordinarily high (Figure 4). Among the 42 families and 120 genera from Xisha Islands in this study, 20 families and 103 genera contain only one species. Therefore, the generally far genetic distances among lineages from the Xisha Islands may interpret why all three DNA barcodes alone have a high rate of species identification. ITS showed a higher rate of species identification in groups with richer species diversity than *rbc*L or *mat*K except for *Digitaria*, of which the five species are morphologically similar and very difficult to be distinguished. Specific DNA barcodes or genomic data may be required to distinguish such closely related species. Considering the trade‐off between primer universality and species discriminatory power, we recommend ITS as the DNA barcode for the flowering plants of Xisha Islands and other oceanic coral islets. This study provides the first DNA barcode data of the flora from the tropical oceanic coral islands. The results indicate that ITS shows the highest rate of species resolution for single DNA barcode with an excellent primer universality. ITS could be the best choice for barcoding the flowering plants from Xisha Islands since the three‐barcode combination (*rbc*L + matK + ITS) didn't significantly improve the species resolution. However, the two plastid markers could be informative in addressing questions regarding community ecology and the evolutionary relations among community members. The two plastid markers could also help people to understand the relationships of co‐occurring species and the species assembly within community when combining more information including species abundance on each island and the functional traits of all the species in the future. In addition, the taxonomic structure of flowering plants on Xisha Islands indicates that biodiversity loss could be more likely to happen on tropical coral islands. As a result, we need to pay more attention to the biodiversity conservation in this unique ecosystem. The nucleotide information of the dominant or constructive species (such as *Suriana maritima*,* Guettarda speciose*,* Pisonia grandis*) and the rare and endangered species (such as *Pemphis acidula*,* Eulophia graminea* Lindl. [Orchidaceae]) in this study may be of significance for ecological restoration and species conservation.

## CONFLICT OF INTEREST

None declared.

## AUTHOR CONTRIBUTIONS

T.T., D.Z, and S.L. conceived the ideas; T.T., S.L., X.Q., X.C., X.L., and J.L. conducted the flora exploring and taxa sampling; S.L. and Z.Z performed the experiments of PCR, and S.L. analyzed the data; S.L. wrote the first draft of the manuscript; S.L., M.S., T.T., and D.Z. contributed to finalizing of the manuscript.

## DATA ACCESSIBILITY

A list of the plant samples and their GenBank accession details are provided in Supporting Information Table S1.

## References

[ece34545-bib-0001] Altschul, S. F. , Gish, W. , Miller, W. , Myers, E. W. , & Lipman, D. J. (1990). Basic local alignment search tool. Journal of Molecular Biology, 215, 403–410. 10.1016/S0022-2836(05)80360-2 2231712

[ece34545-bib-0002] Alvarez, I. , & Wendel, J. F. (2003). Ribosomal ITS sequences and plant phylogenetic inference. Molecular Phylogenetics and Evolution, 29, 417–434. 10.1016/S1055-7903(03)00208-2 14615184

[ece34545-bib-0003] Baldwin, B. G. , Sanderson, M. J. , Porter, J. M. , Wojciechowski, M. F. , Campbell, C. S. , & Donoghue, M. J. (1995). The ITS region of nuclear ribosomal DNA: A valuable source of evidence on angiosperm phylogeny. Annals of the Missouri Botanical Garden, 82, 247–277. 10.2307/2399880

[ece34545-bib-0004] Burgess, K. S. , Fazekas, A. J. , Kesanakurti, P. R. , Graham, S. W. , Husband, B. C. , Newmaster, S. G. , … Barrett, S. C. H. (2011). Discriminating plant species in a local temperate flora using the *rbc*L + *mat*K DNA barcode. Methods in Ecology and Evolution, 2(4), 333–340. 10.1111/j.2041-210X.2011.00092.x

[ece34545-bib-0005] CBOL Plant Working Group (2009). A DNA barcode for land plants. Proceedings of the National Academy of Sciences of the United States of America, 106(31), 12794–12797. 10.1073/pnas.0905845106 19666622 PMC2722355

[ece34545-bib-0006] Chen, S. , Yao, H. , Han, J. , Song, J. , Zhu, Y. , Ma, X. , … Leon, C. (2010). Validation of the ITS2 region as a novel DNA barcode for identifying medicinal plant species. PLoS One, 5, e8613. 20062805 10.1371/journal.pone.0008613 PMC2799520

[ece34545-bib-0007] Costion, C. , Ford, A. , Cross, H. , Crayn, D. , Harrington, M. , & Lowe, A. (2011). Plant DNA barcodes can accurately estimate species richness in poorly known floras. PLoS One, 6(11), e26841 10.1371/journal.pone.0026841 22096501 PMC3214028

[ece34545-bib-0008] Cuénoud, P. , Savolainen, V. , Chatrou, L. W. , Powell, M. , Grayer, R. J. , & Chase, M. W. (2002). Molecular phylogenetics of Caryophyllales based on nuclear 18S rDNA and plastid *rbc*L, *atp*B, and *mat*K DNA sequences. American Journal of Botany, 89, 132–144. 10.3732/ajb.89.1.132 21669721

[ece34545-bib-0009] Cullings, K. W. , & Vogler, D. R. (1998). A 5.8S nuclear ribosomal RNA gene sequence database. Molecular Ecology, 7, 919–923. 9691493 10.1046/j.1365-294x.1998.00409.x

[ece34545-bib-0010] Doyle, J. , & Doyle, J. (1987). A rapid DNA isolation procedure for small quantities of fresh leaf tissue. Phytochemical Bulletin, 19, 11–15.

[ece34545-bib-0011] Faith, D. P. (1992). Conservation evaluation and phylogenetic diversity. Biological Conservation, 61, 1–10. 10.1016/0006-3207(92)91201-3

[ece34545-bib-0012] Faith, D. P. (2008). Phylogenetic diversity and conservation In Carroll S. P., & Fox C. (Eds.), Conservation biology: Evolution in action (pp. 99–115). New York, NY: Oxford University Press Inc.

[ece34545-bib-0013] Fay, M. F. , Swensen, S. M. , & Chase, M. W. (1997). Taxonomic affinities of *Medusagyne oppositifolia* (Medusagynaceae). Kew Bulletin, 52, 111–120. 10.2307/4117844

[ece34545-bib-0014] Fazekas, A. J. , Burgess, K. S. , Kesanakurti, P. R. , Graham, S. W. , Newmaster, S. G. , Husband, B. C. , … Barrett, S. C. H. (2008). Multiple multilocus DNA barcodes from the plastid genome discriminate plant species equally well. PLoS One, 3(7), e2802 10.1371/journal.pone.0002802 18665273 PMC2475660

[ece34545-bib-0015] Ford, C. S. , Ayres, K. L. , Toomey, N. , & Liu, C. (2009). Selection of candidate coding DNA barcoding regions for use on land plants. Botanical Journal of the Linnean Society, 159, 1–11.

[ece34545-bib-0016] Forest, F. , Grenyer, R. , Rouget, M. , Davies, J. , Cowling, R. M. , Faith, D. P. , … Savolainen, V. (2007). Preserving the evolutionary potential of floras in biodiversity hotspots. Nature, 445, 757–760. 10.1038/nature05587 17301791

[ece34545-bib-0017] García‐Robledo, C. , Kuprewicz, E. K. , Staines, C. L. , Kress, W. J. , & Erwin, T. L. (2013). Using a comprehensive DNA barcode library to detect novel egg and larval host plant associations in *Cephaloleia* rolled‐leaf beetles (Coleoptera: Chrysomelidae). Biological Journal of the Linnean Society, 110, 189–198.

[ece34545-bib-0018] Gonzalez, M. A. , Baraloto, C. , Engel, J. , Mori, S. A. , Pétronelli, P. , Riéra, B. , … Chave, J. (2009). Identification of Amazonian trees with DNA barcodes. PLoS One, 4(10), e7483 10.1371/journal.pone.0007483 19834612 PMC2759516

[ece34545-bib-0019] Gregory, T. R. (2005). DNA barcoding does not compete with taxonomy. Nature, 434, 1067. 10.1038/4341067b 15858548

[ece34545-bib-0020] Hamsher, S. E. , & Saunders, G. W. (2014). A floristic survey of marine tube‐forming diatoms reveals unexpected diversity and extensive co‐habitation among genetic lines of the *Berkeleya rutilans* complex (Bacillariophyceae). European Journal of Phycology, 49, 47–59.

[ece34545-bib-0021] Hebert, P. D. N. , Penton, E. H. , Burns, J. M. , Janzen, D. H. , & Hallwachs, W. (2004). Ten species in one: DNA barcoding reveals cryptic species in the neotropical skipper butterfly *Astraptes fulgerator* . Proceedings of the National Academy of Sciences of the United States of America, 101, 14812–14817. 10.1073/pnas.0406166101 15465915 PMC522015

[ece34545-bib-0022] Hollingsworth, P. M. , Graham, S. W. , & Little, D. P. (2011). Choosing and using a plant DNA barcode. PLoS One, 6, e19254 10.1371/journal.pone.0019254 21637336 PMC3102656

[ece34545-bib-0023] Kearse, M. , Moir, R. , Wilson, A. , Stones‐Havas, S. , Cheung, M. , Sturrock, S. , … Drummond, A. (2012). Geneious basic: An integrated and extendable desktop software platform for the organization and analysis of sequence data. Bioinformatics, 28(12), 1647–1649. 10.1093/bioinformatics/bts199 22543367 PMC3371832

[ece34545-bib-0024] Kress, W. J. , & Erickson, D. L. (2007). A two–locus global DNA barcode for land plants: The coding *rbc*L gene complements the non–coding *trn*H–*psb*A spacer region. PLoS One, 2(6), e508 10.1371/journal.pone.0000508 17551588 PMC1876818

[ece34545-bib-0025] Kress, W. J. , Erickson, D. L. , Jones, F. A. , Swenson, N. G. , Perez, R. , Sanjur, O. , & Bermingham, E. (2009). Plant DNA barcodes and a community phylogeny of a tropical forest dynamics plot in Panama. Proceedings of the National Academy of Sciences of the United States of America, 106(44), 18621–18626. 10.1073/pnas.0909820106 19841276 PMC2763884

[ece34545-bib-0026] Kress, W. J. , García‐Robledo, C. , Uriarte, M. , & Erickson, D. L. (2015). DNA barcodes for ecology, evolution, and conservation. Trends in Ecology & Evolution, 30, 25–35. 10.1016/j.tree.2014.10.008 25468359

[ece34545-bib-0027] Kress, W. J. , Wurdack, K. J. , Zimmer, E. A. , Weigt, L. A. , & Janzen, D. H. (2005). Use of DNA barcodes to identify flowering plants. Proceedings of the National Academy of Sciences of the United States of America, 102(23), 8369–8374. 10.1073/pnas.0503123102 15928076 PMC1142120

[ece34545-bib-0028] Kuzmina, M. L. , Johnson, K. L. , Barron, H. R. , & Hebert, P. D. (2012). Identification of the vascular plants of Churchill, Manitoba, using a DNA barcode library. BMC Ecology, 12, 25 10.1186/1472-6785-12-25 23190419 PMC3538695

[ece34545-bib-0029] Li, D. Z. , Gao, L. M. , Li, H. T. , Wang, H. , Ge, X. J. , Liu, J. Q. , … Duan, G. W. (2011). Comparative analysis of a large dataset indicates that internal transcribed spacer (ITS) should be incorporated into the core barcode for seed plants. Proceedings of the National Academy of Sciences of the United States of America, 108(49), 19641–19646. 22100737 10.1073/pnas.1104551108 PMC3241788

[ece34545-bib-0030] Little, D. P. , & Barrington, D. S. (2003). Major evolutionary events in the origin and diversification of the fern genus *Polystichum* (Dryopteridaceae). American Journal of Botany, 90, 508–514. 10.3732/ajb.90.3.508 21659143

[ece34545-bib-0031] Liu, J. , Yan, H. F. , Newmaster, S. G. , Pei, N. C. , Ragupathy, S. , & Ge, X. J. (2015). The use of DNA barcoding as a tool for the conservation biogeography of subtropical forests in China. Diversity and Distributions, 21, 188–199. 10.1111/ddi.12276

[ece34545-bib-0032] Merckx, V. F. T. , Hendriks, K. , Beentjes, K. , Mennes, C. , Becking, L. , Peijnenburg, K. , … Jocqué, M. (2015). Evolution of endemism on a young tropical mountain. Nature, 524, 347–350. 10.1038/nature14949 26266979

[ece34545-bib-0033] Monaghan, M. T. , Balke, M. , Pons, J. , & Vogler, A. P. (2006). Beyond barcodes: Complex DNA taxonomy of a South Pacific Island radiation. Proceedings of the Royal Society of London. Series B, Biological Sciences, 273, 887–893. 10.1098/rspb.2005.3391 16618684 PMC1560222

[ece34545-bib-0034] Nieto Feliner, G. , & Rosselló, J. A. (2007). Better the devil you know? Guidelines for insightful utilization of nrDNA ITS in species‐level evolutionary studies in plants. Molecular Phylogenetics and Evolution, 44, 911–919. 10.1016/j.ympev.2007.01.013 17383902

[ece34545-bib-0035] Pagel, M. (1999). Inferring the historical patterns of biological evolution. Nature, 401, 877–884. 10.1038/44766 10553904

[ece34545-bib-0036] Parmentier, I. , Duminil, J. , Kuzmina, M. , Philippe, M. , Thomas, D. W. , Kenfack, D. , … Hardy, O. J. (2013). How effective are DNA barcodes in the identification of African rainforest trees? PLoS One, 8(4), e54921 10.1371/journal.pone.0054921 23565134 PMC3615068

[ece34545-bib-0037] Pei, N. C. , Erickson, D. L. , Chen, B. F. , Ge, X. J. , Mi, X. C. , Swenson, N. G. , … Kress, W. J. (2015). Closely‐related taxa influence woody species discrimination via DNA barcoding: Evidence from global forest dynamics plots. Scientific Reports, 5, 15127 10.1038/srep15127 26456472 PMC4601009

[ece34545-bib-0038] Purvis, A. , & Hector, A. (2000). Getting the measure of biodiversity. Nature, 405, 212–219. 10.1038/35012221 10821281

[ece34545-bib-0039] Saarela, M. , Sokoloff, P. C. , Gillespie, L. J. , Consaul, L. L. , & Bull, R. D. (2013). DNA barcoding the Canadian arctic flora: Core plastid barcodes (*rbc*L + *mat*K) for 490 vascular plant species. PLoS One, 8(10), e77982 10.1371/journal.pone.0077982 24348895 PMC3865322

[ece34545-bib-0040] Sass, C. , Little, D. P. , Stevenson, D. W. , & Specht, C. D. (2007). DNA barcoding in the Cycadales: Testing the potential of proposed barcoding markers for species identification of cycads. PLoS One, 2, e1154 10.1371/journal.pone.0001154 17987130 PMC2063462

[ece34545-bib-0041] Silva, E. D. , de Abreu, C. B. , Orlando, T. C. , Wisniewski, C. , & Santos‐Wisniewski, M. J. D. (2014). *Alona iheringula* Sinev & Kotov, 2004 (Crustacea, Anomopoda, Chydoridae, Aloninae): Life cycle and DNA barcode with implications for the taxonomy of the Aloninae subfamily. PLoS One, 9, e97050. 24878503 10.1371/journal.pone.0097050 PMC4039433

[ece34545-bib-0042] Smith, M. A. , Fernández‐Triana, J. L. , Eveleigh, E. , Gómez, J. , Guclu, C. , Hallwachs, W. , … Ar‐Riverón, A. Z. (2012). DNA barcoding and the taxonomy of Microgastrinae wasps (Hymenoptera, Braconidae): Impacts after 8 years and nearly 20000 sequences. Molecular Ecology Resources, 13, 168–176. 23228011 10.1111/1755-0998.12038

[ece34545-bib-0043] Suh, Y. , Thien, L. B. , Reeve, H. E. , & Zimmer, E. A. (1996). Molecular evolution and phylogenetic implication of internal transcribed spacer sequences of ribosomal DNA in Winteraceae. American Journal of Botany, 80, 1042–1055.

[ece34545-bib-0044] Swofford, D. L. (2000). PAUP*: Phylogenetic analysis using parsimony (*and other methods), version 4.0b. Sunderland, MA: Sinauer Associates.

[ece34545-bib-0045] Telfer, A. C. , Young, M. R. , Quinn, J. , Perez, K. , Sobel, C. N. , Sones, J. E. , … deWaard, J. R. (2015). Biodiversity inventories in high gear: DNA barcoding facilitates a rapid biotic survey of a temperate nature reserve. Biodiversity Data Journal, 3, e6313 10.3897/BDJ.3.e6313 PMC4568406 26379469

[ece34545-bib-0046] Theodoridis, S. , Stefanaki, A. , Tezcan, M. , Aki, C. , Kokkini, S. , & Vlachonasios, K. E. (2012). DNA barcoding in native plants of the Labiatae (Lamiaceae) family from Chios Island (Greece) and the adjacent Cesme‐Karaburun Peninsula (Turkey). Molecular Ecology Resources, 12, 620–633. 22394710 10.1111/j.1755-0998.2012.03129.x

[ece34545-bib-0047] Tong, Y. , Jian, S. G. , Chen, Q. , Li, Y. L. , & Xing, F. W. (2013). Vascular plant diversity of the Paracel Islands, China. Biodiversity Science, 21(3), 364–374.

[ece34545-bib-0048] Webb, C. O. , Ackerly, D. D. , McPeek, M. A. , & Donoghue, M. J. (2002). Phylogenies and community ecology. Annual Review of Ecology and Systematics, 33, 475–505. 10.1146/annurev.ecolsys.33.010802.150448

[ece34545-bib-0049] Wen, J. , & Zimmer, E. A. (1996). Phylogeny and biogeography of *Panax* L. (the Ginseng genus, Araliaceae): Inferences from ITS sequences of nuclear ribosomal DNA. Molecular Phylogenetics and Evolution, 6, 167–177. 8899721 10.1006/mpev.1996.0069

[ece34545-bib-0050] Winterbottom, R. , Hanner, R. H. , Burridge, M. , & Zur, M. (2014). A cornucopia of cryptic species: A DNA barcode analysis of the gobiid fish genus *Trimma* (Percomorpha, Gobiiformes). ZooKeys, 381, 79–111. 10.3897/zookeys.381.6445 PMC3950426 24624015

[ece34545-bib-0051] Wu, Z. Y. , Raven, P. H. , & Hong, D. Y. (Eds.) (1994–2013). Flora of China. Beijing, China: Science Press, and St. Louis, MO: Missouri Botanical Garden Press.

[ece34545-bib-0052] Yao, H. , Song, J. , Liu, C. , Luo, K. , Han, J. , Li, Y. , … Chen, S. L. (2010). Use of ITS2 region as the universal DNA barcode for plants and animals. PLoS One, 5, e13102. 20957043 10.1371/journal.pone.0013102 PMC2948509

[ece34545-bib-0053] Zhang, W. , Wendel, J. F. , & Clark, L. G. (1997). Bamboozled again! Inadvertent isolation of fungal rDNA sequences from bamboos (Poaceae: Bambusoideae). Molecular Phylogenetics and Evolution, 8, 205–217. 10.1006/mpev.1997.0422 9299225

