# Supplementary material for: DNA barcoding the flowering plants from the tropical coral islands of Xisha (China)
Source: Ecol Evol. 2018 Oct 3;8(21):10587–93. doi: 10.1002/ece3.4545 (PMC6238132; doi:10.1002/ece3.4545)
Supplement: Supplementary file 1 [file ECE3-8-10587-s001.docx]

**Supporting information**

Table S1. List of taxa, vouchers, and GenBank accession numbers.

| Taxon | Vouchers | *rbcL* | *matK* | ITS |
| --- | --- | --- | --- | --- |
| *Agave sisalana* Perrine | CHB12270 | n/a | n/a | MH768059 |
| *Agave sisalana* Perrine | LSC175 | n/a | n/a | MH768060 |
| *Sesuvium portulacastrum* (L.) L. | TuTY1396 | MH767453 | MH767761 | MH768061 |
| *Sesuvium portulacastrum* (L.) L. | TuTY1514 | MH767454 | MH767762 | MH768062 |
| *Trianthema portulacastrum* L. | LSC59 | MH767455 | MH767763 | MH768063 |
| *Trianthema portulacastrum* L. | TuTY1465 | MH767456 | MH767764 | MH768064 |
| *Achyranthes aspera* L. | TuTY1518 | MH767457 | MH767765 | MH768065 |
| *Achyranthes aspera* L. | TuTY2243 | MH767458 | MH767766 | MH768066 |
| *Amaranthus viridis* L. | ZLX12377 | MH767459 | MH767767 | MH768067 |
| *Amaranthus viridis* L. | TuTY1472 | MH767460 | MH767768 | MH768068 |
| *Celosia argentea* L. | TuTY1478 | MH767461 | MH767769 | MH768069 |
| *Celosia argentea* L. | TuTY2247 | MH767462 | MH767770 | MH768070 |
| *Cordia subcordata* Lam. | TuTY1447 | MH767463 | MH767771 | MH768071 |
| *Cordia subcordata* Lam. | TuTY2071 | MH767464 | MH767772 | MH768072 |
| *Heliotropium indicum* L. | LSC98 | MH767465 | MH767773 | MH768073 |
| *Heliotropium indicum* L. | TuTY1446 | MH767466 | MH767774 | MH768074 |
| *Tournefortia argentea* L. f. | TuTY1384 | MH767467 | MH767775 | MH768075 |
| *Tournefortia argentea* L. f. | TuTY1580 | MH767468 | MH767776 | MH768076 |
| *Tournefortia argentea* L. f. | TuTY2115 | MH767469 | MH767777 | MH768077 |
| *Caesalpinia bonduc* (L.) Roxb. | TuTY2139 | MH767470 | MH767778 | MH768078 |
| *Caesalpinia bonduc* (L.) Roxb. | TuTY2615xs | MH767471 | MH767779 | MH768079 |
| *Chamaecrista pumila* (Lam.) K.Larsen | LSC125 | MH767472 | MH767780 | MH768080 |
| *Senna occidentalis* (L.) Link | LSC18 | MH767473 | MH767781 | MH768081 |
| *Senna occidentalis* (L.) Link | TuTY1498 | MH767474 | MH767782 | MH768082 |
| *Senna occidentalis* (L.) Link | TuTY2256 | MH767475 | MH767783 | MH768083 |
| *Cleome gynandra* L. | LSC76 | MH767476 | MH767784 | MH768084 |
| *Cleome gynandra* L. | TuTY1439 | MH767477 | MH767785 | MH768085 |
| *Cleome rutidosperma* DC. | LSC8 | MH767478 | MH767786 | MH768086 |
| *Cleome rutidosperma* DC. | TuTY1459 | MH767479 | MH767787 | MH768087 |
| *Cleome viscosa* L. | TuTY1386 | MH767480 | MH767788 | MH768088 |
| *Cleome viscosa* L. | TuTY2203 | MH767481 | MH767789 | MH768089 |
| *Chenopodium acuminatum* Willd. | TuTY1507 | MH767482 | MH767790 | MH768090 |
| *Terminalia catappa* L. | TuTY1409 | MH767483 | MH767791 | MH768091 |
| *Terminalia catappa* L. | TuTY1415 | MH767484 | MH767792 | MH768092 |
| *Commelina benghalensis* L. | LSC75 | MH767485 | MH767793 | n/a |
| *Commelina benghalensis* L. | TuTY1395 | MH767486 | MH767794 | MH768093 |
| *Commelina benghalensis* L. | TuTY2299 | MH767487 | MH767795 | MH768094 |
| *Bidens pilosa* L. | LSC107 | MH767488 | MH767796 | MH768095 |
| *Bidens pilosa* L. | TuTY2689 | MH767489 | MH767797 | MH768096 |
| *Chromolaena odorata* (L.) R.M.King & H.Rob. | TuTY1422 | MH767490 | MH767798 | MH768097 |
| *Chromolaena odorata* (L.) R.M.King & H.Rob. | TuTY2696 | MH767491 | MH767799 | MH768098 |
| *Cyanthillium cinereum* (L.) H.Rob. | TuTY1469 | MH767492 | MH767800 | MH768099 |
| *Cyanthillium cinereum* (L.) H.Rob. | TuTY2199 | MH767493 | MH767801 | MH768100 |
| *Eclipta prostrata* (L.) L. | TuTY1479 | MH767494 | MH767802 | MH768101 |
| *Eclipta prostrata* (L.) L. | ZLX12429 | MH767495 | MH767803 | MH768102 |
| *Emilia sonchifolia* (L.) DC. ex DC. | LSC90 | MH767496 | MH767804 | MH768103 |
| *Emilia sonchifolia* (L.) DC. ex DC. | TuTY1529 | MH767497 | MH767805 | MH768104 |
| *Erigeron canadensis* L. | TuTY1527 | MH767498 | MH767806 | MH768105 |
| *Laphangium affine* (D.Don) Tzvelev | TuTY1496 | MH767499 | MH767807 | MH768106 |
| *Launaea sarmentosa* (Willd.) Sch.Bip. ex Kuntze | LSC135 | MH767500 | MH767808 | MH768107 |
| *Launaea sarmentosa* (Willd.) Sch.Bip. ex Kuntze | TuTY1526 | MH767501 | MH767809 | MH768108 |
| *Praxelis clematidea* (Griseb.) R.M.King & H.Rob. | LSC122 | MH767502 | MH767810 | MH768109 |
| *Praxelis clematidea* (Griseb.) R.M.King & H.Rob. | TuTY1520 | MH767503 | MH767811 | MH768110 |
| *Sphagneticola trilobata* (L.) Pruski | TuTY1524 | MH767504 | MH767812 | MH768111 |
| *Sphagneticola trilobata* (L.) Pruski | ZLX12333 | MH767505 | MH767813 | MH768112 |
| *Tridax procumbens* (L.) L. | TuTY1424 | MH767506 | MH767814 | MH768113 |
| *Tridax procumbens* (L.) L. | TuTY2202 | MH767507 | MH767815 | MH768114 |
| *Wollastonia biflora* (L.) DC. | TuTY1404 | MH767508 | MH767816 | MH768115 |
| *Wollastonia biflora* (L.) DC. | TuTY1576 | MH767509 | n/a | MH768116 |
| *Evolvulus alsinoides* (L.) L. | HXX18284 | MH767510 | MH767817 | MH768117 |
| *Evolvulus alsinoides* (L.) L. | TuTY2085 | MH767511 | MH767818 | MH768118 |
| *Ipomoea indica* (Burm.) Merr. | LSC117 | MH767512 | MH767819 | MH768119 |
| *Ipomoea indica* (Burm.) Merr. | LSC87 | MH767513 | MH767820 | MH768120 |
| *Ipomoea obscura* (L.) Ker Gawl. | TuTY1511 | MH767514 | MH767821 | MH768121 |
| *Ipomoea obscura* (L.) Ker Gawl. | TuTY2296 | MH767515 | MH767822 | MH768122 |
| *Ipomoea pes-caprae* (L.) R. Br. | TuTY1405 | MH767516 | MH767823 | MH768123 |
| *Ipomoea pes-caprae* (L.) R. Br. | TuTY1515 | MH767517 | MH767824 | MH768124 |
| *Ipomoea pes-tigridis* L. | LSC77 | MH767518 | MH767825 | MH768125 |
| *Ipomoea pes-tigridis* L. | ZLX12357 | MH767519 | MH767826 | MH768126 |
| *Ipomoea violacea* L. | TuTY1397 | MH767520 | MH767827 | MH768127 |
| *Ipomoea violacea* L. | TuTY1460 | MH767521 | MH767828 | MH768128 |
| *Ipomoea violacea* L. | TuTY1594 | MH767522 | MH767829 | MH768129 |
| *Merremia tridentata* (L.) Hallier f. | TuTY2169 | MH767523 | MH767830 | MH768130 |
| *Coccinia grandis* (L.) Voigt | LSC74 | MH767524 | MH767831 | n/a |
| *Coccinia grandis* (L.) Voigt | TuTY1438 | MH767525 | MH767832 | n/a |
| *Cyperus compressus* L. | TuTY2068 | MH767526 | n/a | MH768131 |
| *Cyperus javanicus* Houtt. | TuTY2245 | MH767527 | MH767833 | n/a |
| *Cyperus rotundus* L. | LSC41 | MH767528 | MH767834 | MH768132 |
| *Cyperus rotundus* L. | TuTY2230 | MH767529 | MH767835 | n/a |
| *Cyperus stoloniferus* Retz. | LSC40 | MH767530 | n/a | MH768133 |
| *Cyperus stoloniferus* Retz. | TuTY2264 | MH767531 | MH767836 | MH768134 |
| *Fimbristylis cymosa var. spathacea* (Roth) T.Koyama | LSC136 | MH767532 | MH767837 | MH768135 |
| *Fimbristylis cymosa var. spathacea* (Roth) T.Koyama | TuTY2205 | MH767533 | MH767838 | MH768136 |
| *Acalypha lanceolata* Willd. | LSC58 | MH767534 | MH767839 | MH768137 |
| *Acalypha lanceolata* Willd. | TuTY1467 | MH767535 | MH767840 | MH768138 |
| *Euphorbia atoto* G.Forst. | LSC71 | MH767536 | MH767841 | MH768139 |
| *Euphorbia atoto* G.Forst. | TuTY1412 | MH767537 | MH767842 | MH768140 |
| *Euphorbia atoto* G.Forst. | TuTY1510 | MH767538 | MH767843 | MH768141 |
| *Euphorbia cyathophora* Murray | LSC34 | MH767539 | MH767844 | MH768142 |
| *Euphorbia cyathophora* Murray | TuTY1463 | MH767540 | MH767845 | MH768143 |
| *Euphorbia hirta* L. | TuTY1400 | MH767541 | MH767846 | MH768144 |
| *Euphorbia hirta* L. | TuTY2270 | MH767542 | MH767847 | MH768145 |
| *Euphorbia prostrata* Aiton | TuTY1408 | MH767543 | MH767848 | MH768146 |
| *Euphorbia prostrata* Aiton | ZLX12400 | MH767544 | MH767849 | MH768147 |
| *Euphorbia thymifolia* L. | LSC51 | MH767545 | MH767850 | MH768148 |
| *Euphorbia thymifolia* L. | TuTY2222 | MH767546 | MH767851 | MH768149 |
| *Euphorbia thymifolia* L. | TuTY2525 | MH767547 | MH767852 | MH768150 |
| *Micrococca mercurialis* (L.) Benth. | LSC12 | MH767548 | MH767853 | MH768151 |
| *Micrococca mercurialis* (L.) Benth. | TuTY1497 | MH767549 | MH767854 | MH768152 |
| *Micrococca mercurialis* (L.) Benth. | TuTY2244 | MH767550 | MH767855 | MH768153 |
| *Microstachys chamaelea* (L.) Müll.Arg. | CBH11809 | MH767551 | MH767856 | MH768154 |
| *Microstachys chamaelea* (L.) Müll.Arg. | TuTY2116 | MH767552 | MH767857 | MH768155 |
| *Phyllanthus niruri* L. | TuTY1468 | MH767553 | MH767858 | MH768156 |
| *Phyllanthus niruri* L. | TuTY2211 | MH767554 | n/a | MH768157 |
| *Phyllanthus virgatus* G.Forst. | TuTY2121 | MH767555 | MH767859 | MH768158 |
| *Phyllanthus virgatus* G.Forst. | TuTY2174 | MH767556 | MH767860 | MH768159 |
| *Ricinus communis* L. | LSC45 | MH767557 | MH767861 | MH768160 |
| *Ricinus communis* L. | TuTY1421 | MH767558 | MH767862 | MH768161 |
| *Sauropus bacciformis* (L.) Airy Shaw | ZLX12430 | MH767559 | MH767863 | MH768162 |
| *Sauropus bacciformis* (L.) Airy Shaw | CBH11834 | MH767560 | MH767864 | MH768163 |
| *Scaevola taccada* (Gaertn.) Roxb. | TuTY1392 | MH767561 | MH767865 | MH768164 |
| *Scaevola taccada* (Gaertn.) Roxb. | TuTY1570 | MH767562 | MH767866 | MH768165 |
| *Bothriochloa ischaemum* (L.) Keng | TuTY1522 | MH767563 | MH767867 | MH768166 |
| *Bothriochloa ischaemum* (L.) Keng | TuTY2250 | MH767564 | MH767868 | MH768167 |
| *Brachiaria ramosa* (L.) Stapf | LSC124 | MH767565 | MH767869 | MH768168 |
| *Brachiaria ramosa* (L.) Stapf | TuTY2237 | MH767566 | MH767870 | MH768169 |
| *Brachiaria subquadripara* (Trin.) Hitchc. | TuTY1445 | MH767567 | MH767871 | MH768170 |
| *Brachiaria subquadripara* (Trin.) Hitchc. | TuTY2158 | MH767568 | MH767872 | MH768171 |
| *Brachiaria subquadripara* (Trin.) Hitchc. | TuTY2219 | MH767569 | MH767873 | MH768172 |
| *Brachiaria subquadripara* (Trin.) Hitchc. | TuTY2224 | MH767570 | n/a | MH768173 |
| *Cenchrus echinatus* L. | TuTY1456 | MH767571 | MH767874 | MH768174 |
| *Cenchrus echinatus* L. | TuTY2278 | MH767572 | MH767875 | MH768175 |
| *Chloris formosana* (Honda) Keng | LSC65 | MH767573 | MH767876 | MH768176 |
| *Chloris formosana* (Honda) Keng | TuTY2053 | MH767574 | MH767877 | MH768177 |
| *Cynodon dactylon* (L.) Pers. | LSC63 | MH767575 | MH767878 | MH768178 |
| *Cynodon dactylon* (L.) Pers. | TuTY1454 | MH767576 | MH767879 | MH768179 |
| *Cynodon dactylon* (L.) Pers. | TuTY2252 | MH767577 | MH767880 | MH768180 |
| *Dactyloctenium aegyptium* (L.) Willd. | CBH12193 | MH767578 | MH767881 | MH768181 |
| *Dactyloctenium aegyptium* (L.) Willd. | TuTY2043 | MH767579 | MH767882 | MH768182 |
| *Digitaria ciliaris* (Retz.) Koeler | TuTY2168 | MH767580 | MH767883 | n/a |
| *Digitaria heterantha* (Hook.f.) Merr. | LSC126 | MH767581 | MH767884 | MH768183 |
| *Digitaria heterantha* (Hook.f.) Merr. | TuTY2254 | MH767582 | MH767885 | MH768184 |
| *Digitaria radicosa* (J.Presl) Miq. | TuTY1403 | MH767583 | MH767886 | MH768185 |
| *Digitaria radicosa* (J.Presl) Miq. | TuTY2171 | MH767584 | MH767887 | MH768186 |
| *Digitaria sanguinalis* (L.) Scop. | TuTY2258 | MH767585 | MH767888 | MH768187 |
| *Digitaria setigera* Roth | TuTY2047 | MH767586 | MH767889 | n/a |
| *Digitaria setigera* Roth | TuTY2067 | MH767587 | MH767890 | MH768188 |
| *Eleusine indica* (L.) Gaertn. | TuTY1406 | MH767588 | MH767891 | MH768189 |
| *Eleusine indica* (L.) Gaertn. | TuTY2061 | MH767589 | MH767892 | MH768190 |
| *Eragrostis amabilis* (L.) Wight & Arn. | TuTY1451 | MH767590 | MH767893 | MH768191 |
| *Eragrostis amabilis* (L.) Wight & Arn. | TuTY2255 | MH767591 | MH767894 | MH768192 |
| *Eriochloa procera* (Retz.) C.E.Hubb. | LSC66 | MH767592 | MH767895 | MH768193 |
| *Eriochloa procera* (Retz.) C.E.Hubb. | TuTY2091 | MH767593 | MH767896 | MH768194 |
| *Eriochloa procera* (Retz.) C.E.Hubb. | TuTY2661xs | MH767594 | MH767897 | MH768195 |
| *Heteropogon contortus* (L.) P.Beauv. ex Roem. & Schult. | LSC101 | MH767595 | n/a | MH768196 |
| *Heteropogon contortus* (L.) P.Beauv. ex Roem. & Schult. | TuTY1505 | MH767596 | MH767898 | MH768197 |
| *Heteropogon contortus* (L.) P.Beauv. ex Roem. & Schult. | TuTY2297 | MH767597 | MH767899 | MH768198 |
| *Imperata cylindrica* (L.) Raeusch. | CBH12259 | MH767598 | MH767900 | MH768199 |
| *Imperata cylindrica* (L.) Raeusch. | TuTY2304 | MH767599 | MH767901 | MH768200 |
| *Lepturus repens* (J.R.Forst.) R.Br. | TuTY1402 | MH767600 | MH767902 | MH768201 |
| *Lepturus repens* (J.R.Forst.) R.Br. | TuTY1426 | MH767601 | MH767903 | MH768202 |
| *Melinis repens* (Willd.) Zizka | LSC139 | MH767602 | MH767904 | MH768203 |
| *Melinis repens* (Willd.) Zizka | ZLX12439 | MH767603 | MH767905 | MH768204 |
| *Panicum repens* L. | CBH12194 | MH767604 | MH767906 | MH768205 |
| *Panicum repens* L. | TuTY2235 | MH767605 | MH767907 | MH768206 |
| *Paspalum scrobiculatum* L. | TuTY2134 | MH767606 | MH767908 | MH768207 |
| *Sporobolus fertilis* (Steud.) Clayton | TuTY1523 | MH767607 | MH767909 | MH768208 |
| *Sporobolus fertilis* (Steud.) Clayton | TuTY2240 | MH767608 | MH767910 | MH768209 |
| *Sporobolus virginicus* (L.) Kunth | LSC38 | MH767609 | MH767911 | MH768210 |
| *Stenotaphrum micranthum* (Desv.) C.E.Hubb. | TuTY1541 | MH767610 | n/a | MH768211 |
| *Stenotaphrum micranthum* (Desv.) C.E.Hubb. | TuTY1572 | MH767611 | MH767912 | MH768212 |
| *Stenotaphrum micranthum* (Desv.) C.E.Hubb. | TuTY1599 | MH767612 | MH767913 | MH768213 |
| *Stenotaphrum micranthum* (Desv.) C.E.Hubb. | TuTY2678xs | MH767613 | MH767914 | MH768214 |
| *Thuarea involuta* (G.Forst.) R.Br. ex Sm. | LSC44 | MH767614 | MH767915 | MH768215 |
| *Thuarea involuta* (G.Forst.) R.Br. ex Sm. | TuTY1532 | MH767615 | MH767916 | MH768216 |
| *Thuarea involuta* (G.Forst.) R.Br. ex Sm. | TuTY2275 | MH767616 | MH767917 | MH768217 |
| *Hyptis suaveolens* (L.) Poit. | TuTY1509 | MH767617 | MH767918 | MH768218 |
| *Leucas chinensis* (Retz.) Sm. | HXX18325 | MH767618 | MH767919 | MH768219 |
| *Leucas chinensis* (Retz.) Sm. | TuTY2132 | MH767619 | MH767920 | MH768220 |
| *Leucas zeylanica* (L.) W.T.Aiton | LSC89 | MH767620 | MH767921 | n/a |
| *Leucas zeylanica* (L.) W.T.Aiton | TuTY1491 | MH767621 | MH767922 | n/a |
| *Cassytha filiformis* L. | TuTY1513 | MH767622 | MH767923 | n/a |
| *Cassytha filiformis* L. | TuTY2184 | MH767623 | MH767924 | n/a |
| *Pemphis acidula* J.R. Forst. & G. Forst. | LSC172 | MH767624 | MH767925 | MH768221 |
| *Pemphis acidula* J.R. Forst. & G. Forst. | TuTY1401 | MH767625 | MH767926 | MH768222 |
| *Pemphis acidula* J.R. Forst. & G. Forst. | TuTY2673xs | MH767626 | MH767927 | MH768223 |
| *Abutilon indicum* (L.) Sweet | TuTY1429 | MH767627 | MH767928 | MH768224 |
| *Abutilon indicum* (L.) Sweet | TuTY2217 | MH767628 | MH767929 | MH768225 |
| *Herissantia crispa* (L.) Brizicky | TuTY1442 | MH767629 | MH767930 | MH768226 |
| *Herissantia crispa* (L.) Brizicky | TuTY1546 | MH767630 | MH767931 | MH768227 |
| *Malvastrum coromandelianum* (L.) Garcke | TuTY1434 | MH767631 | MH767932 | MH768228 |
| *Malvastrum coromandelianum* (L.) Garcke | TuTY2221 | MH767632 | MH767933 | MH768229 |
| *Sida acuta* Burm.f. | LSC31 | MH767633 | MH767934 | MH768230 |
| *Sida acuta* Burm.f. | TuTY1544 | MH767634 | MH767935 | MH768231 |
| *Sida chinensis* Retz. | LSC133 | MH767635 | MH767936 | MH768232 |
| *Sida alnifolia var. microphylla* (Cav.) S.Y. Hu | TuTY1506 | MH767636 | MH767937 | MH768233 |
| *Sida cordata* (Burm.f.) Borss.Waalk. | LSC127 | MH767637 | MH767938 | MH768234 |
| *Sida cordata* (Burm.f.) Borss.Waalk. | TuTY1534 | MH767638 | MH767939 | MH768235 |
| *Sida cordifolia* L. | TuTY1443 | MH767639 | MH767940 | MH768236 |
| *Sida cordifolia* L. | TuTY2340 | MH767640 | MH767941 | MH768237 |
| *Sida parvifolia* DC. | LSC134 | MH767641 | MH767942 | MH768238 |
| *Sida parvifolia* DC. | LSC146 | MH767642 | MH767943 | MH768239 |
| *Sida parvifolia* DC. | TuTY1388 | MH767643 | MH767944 | MH768240 |
| *Sida parvifolia* DC. | TuTY1423 | MH767644 | MH767945 | MH768241 |
| *Sida parvifolia* DC. | TuTY1575 | MH767645 | MH767946 | MH768242 |
| *Sida rhombifolia* L. | ZLX12431 | MH767646 | MH767947 | MH768243 |
| *Urena lobata* L. | LSC37 | MH767647 | MH767948 | MH768244 |
| *Urena lobata* L. | TuTY2117 | MH767648 | MH767949 | MH768245 |
| *Stephania longa* Lour. | CBH12006 | MH767649 | MH767950 | MH768246 |
| *Stephania longa* Lour. | LSC149 | MH767650 | MH767951 | MH768247 |
| *Leucaena leucocephala* (Lam.) de Wit | TuTY1503 | MH767651 | MH767952 | MH768248 |
| *Leucaena leucocephala* (Lam.) de Wit | TuTY2534 | MH767652 | MH767953 | n/a |
| *Mimosa diplotricha* Sauvalle | LSC115 | MH767653 | MH767954 | MH768249 |
| *Mimosa diplotricha* Sauvalle | TuTY2179 | MH767654 | MH767955 | MH768250 |
| *Mimosa pudica* L. | LSC67 | MH767655 | MH767956 | n/a |
| *Mimosa pudica* L. | TuTY1486 | MH767656 | MH767957 | n/a |
| *Glinus oppositifolius* (L.) Aug.DC. | TuTY1473 | MH767657 | MH767958 | MH768251 |
| *Glinus oppositifolius* (L.) Aug.DC. | TuTY1508 | MH767658 | MH767959 | MH768252 |
| *Mollugo nudicaulis* Lam*.* | TuTY2103 | MH767659 | MH767960 | MH768253 |
| *Mollugo nudicaulis* Lam | TuTY2181 | MH767660 | MH767961 | MH768254 |
| *Mollugo stricta* L. | TuTY1432 | MH767661 | MH767962 | MH768255 |
| *Mollugo stricta* L. | TuTY1458 | MH767662 | MH767963 | MH768256 |
| *Mollugo stricta* L. | TuTY1533 | MH767663 | MH767964 | MH768257 |
| *Mollugo verticillata* L. | HXX18866 | MH767664 | MH767965 | MH768258 |
| *Mollugo verticillata* L. | TuTY1433 | MH767665 | MH767966 | MH768259 |
| *Boerhavia diffusa* L. | TuTY1394 | MH767666 | MH767967 | MH768260 |
| *Boerhavia diffusa* L. | TuTY1494 | MH767667 | MH767968 | MH768261 |
| *Boerhavia diffusa* L. | uTY2140 | MH767668 | MH767969 | MH768262 |
| *Boerhavia diffusa* L. | TuTY2153 | MH767669 | MH767970 | MH768263 |
| *Boerhavia erecta* L. | TuTY1573 | MH767670 | MH767971 | MH768264 |
| *Boerhavia erecta* L. | TuTY1591 | MH767671 | MH767972 | MH768265 |
| *Pisonia grandis* R. Br. | TuTY1391 | MH767672 | MH767973 | MH768266 |
| *Pisonia grandis* R. Br. | TuTY1452 | MH767673 | MH767974 | MH768267 |
| *Eulophia graminea* Lindl. | TuTY1502 | n/a | MH767975 | MH768268 |
| *Eulophia graminea* Lindl. | TuTY1547 | n/a | MH767976 | MH768269 |
| *Oxalis corniculata* L. | CBH11536 | MH767674 | n/a | MH768270 |
| *Oxalis corniculata* L. | TuTY2198 | MH767675 | MH767977 | MH768271 |
| *Argemone mexicana* L. | TuTY1485 | MH767676 | MH767978 | MH768272 |
| *Alysicarpus vaginalis* (L.) DC. | TuTY1464 | MH767677 | MH767979 | MH768273 |
| *Alysicarpus vaginalis* (L.) DC. | TuTY2200 | MH767678 | MH767980 | MH768274 |
| *Cajanus scarabaeoides* (L.) Thouars | LSC111 | MH767679 | MH767981 | MH768275 |
| *Cajanus scarabaeoides* (L.) Thouars | TuTY2135 | MH767680 | MH767982 | MH768276 |
| *Canavalia rosea* (Sw.) DC. | TuTY1516 | MH767681 | MH767983 | MH768277 |
| *Canavalia rosea* (Sw.) DC. | TuTY2269 | MH767682 | MH767984 | MH768278 |
| *Crotalaria assamica* Benth. | LSC112 | MH767683 | MH767985 | MH768279 |
| *Crotalaria assamica* Benth. | ZLX12460 | MH767684 | MH767986 | MH768280 |
| *Desmodium triflorum* (L.) DC. | LSC142 | MH767685 | MH767987 | MH768281 |
| *Desmodium triflorum* (L.) DC. | TuTY1430 | MH767686 | MH767988 | MH768282 |
| *Indigofera colutea* (Burm.f.) Merr. | LSC132 | MH767687 | MH767989 | MH768283 |
| *Indigofera colutea* (Burm.f.) Merr. | TuTY2253 | MH767688 | MH767990 | MH768284 |
| *Macroptilium atropurpureum* (DC.) Urb. | TuTY1601 | MH767689 | MH767991 | MH768285 |
| *Rhynchosia minima* (L.) DC. | LSC64 | MH767690 | MH767992 | MH768286 |
| *Rhynchosia minima* (L.) DC. | TuTY1531 | MH767691 | MH767993 | MH768287 |
| *Sesbania bispinosa* (Jacq.) W.Wight | TuTY2649xs | MH767692 | MH767994 | MH768288 |
| *Sesbania bispinosa* (Jacq.) W.Wight | ZLX12438 | MH767693 | MH767995 | MH768289 |
| *Sesbania cannabina* (Retz.) Pers. | LSC92 | MH767694 | MH767996 | MH768290 |
| *Sesbania cannabina* (Retz.) Pers. | TuTY1466 | MH767695 | n/a | MH768291 |
| *Sophora tomentosa* L. | TuTY2668xs | MH767696 | MH767997 | MH768292 |
| *Tephrosia luzoniensis* Vogel | LSC131 | MH767697 | MH767998 | MH768293 |
| *Tephrosia luzoniensis* Vogel | LSC95 | MH767698 | MH767999 | MH768294 |
| *Tephrosia luzoniensis* Vogel | TuTY2259 | MH767699 | MH768000 | MH768295 |
| *Tephrosia purpurea* (L.) Pers. | LSC73 | MH767700 | MH768001 | MH768296 |
| *Tephrosia purpurea* (L.) Pers. | TuTY2231 | MH767701 | MH768002 | MH768297 |
| *Vigna marina* (Burm.) Merr. | TuTY1435 | MH767702 | MH768003 | MH768298 |
| *Vigna marina* (Burm.) Merr. | TuTY1537 | MH767703 | MH768004 | MH768299 |
| *Passiflora foetida* L. | TuTY1450 | MH767704 | n/a | MH768300 |
| *Passiflora foetida* L. | TuTY2248 | MH767705 | n/a | MH768301 |
| *Polygonum plebeium* R.Br. | TuTY1484 | MH767706 | MH768005 | MH768302 |
| *Portulaca oleracea* L. | TuTY1393 | MH767707 | MH768006 | MH768303 |
| *Portulaca oleracea* L. | TuTY2178 | MH767708 | MH768007 | MH768304 |
| *Portulaca pilosa* L. | LSC118 | MH767709 | MH768008 | MH768305 |
| *Portulaca pilosa* L. | TuTY2058 | MH767710 | MH768009 | MH768306 |
| *Colubrina asiatica* (L.) Brongn. | TuTY1517 | MH767711 | MH768010 | MH768307 |
| *Colubrina asiatica* (L.) Brongn. | TuTY2295 | MH767712 | MH768011 | MH768308 |
| *Guettarda speciosa* L. | TuTY1385 | MH767713 | MH768012 | MH768309 |
| *Guettarda speciosa* L. | TuTY1457 | MH767714 | MH768013 | MH768310 |
| *Morinda citrifolia* L. | TuTY1595 | MH767715 | MH768014 | MH768311 |
| *Morinda citrifolia* L. | TuTY1436 | MH767716 | MH768015 | MH768312 |
| *Oldenlandia corymbosa* L. | TuTY1444 | MH767717 | MH768016 | MH768313 |
| *Oldenlandia corymbosa* L. | TuTY2059 | MH767718 | MH768017 | MH768314 |
| *Richardia scabra* L. | TuTY2054 | MH767719 | MH768018 | MH768315 |
| *Richardia scabra* L. | ZLX12433 | MH767720 | MH768019 | MH768316 |
| *Spermacoce hispida* L. | TuTY2118 | MH767721 | MH768020 | MH768317 |
| *Spermacoce pusilla* Wall. | TuTY1475 | MH767722 | MH768021 | MH768318 |
| *Spermacoce pusilla* Wall. | TuTY2208 | MH767723 | MH768022 | MH768319 |
| *Cardiospermum halicacabum* L. | TuTY1495 | MH767724 | MH768023 | MH768320 |
| *Lindernia crustacea* (L.) F.Muell. | LSC160 | MH767725 | MH768024 | n/a |
| *Lindernia crustacea* (L.) F.Muell. | TuTY1481 | MH767726 | MH768025 | n/a |
| *Datura metel* L. | LSC28 | MH767727 | MH768026 | MH768321 |
| *Datura metel* L. | TuTY1419 | MH767728 | MH768027 | MH768322 |
| *Physalis minima* L. | LSC46 | MH767729 | MH768028 | MH768323 |
| *Physalis minima* L. | TuTY2272 | MH767730 | MH768029 | MH768324 |
| *Solanum americanum* Mill. | TuTY1407 | MH767731 | MH768030 | MH768325 |
| *Solanum americanum* Mill. | TuTY1448 | MH767732 | MH768031 | MH768326 |
| *Solanum americanum* Mill. | TuTY2070 | MH767733 | MH768032 | MH768327 |
| *Melochia corchorifolia* L. | TuTY2122 | MH767734 | MH768033 | MH768328 |
| *Melochia corchorifolia* L. | TuTY2173 | MH767735 | MH768034 | MH768329 |
| *Waltheria indica* L. | LSC114 | MH767736 | MH768035 | MH768330 |
| *Waltheria indica* L. | TuTY1455 | MH767737 | MH768036 | MH768331 |
| *Suriana maritima* L. | TuTY1410 | MH767738 | MH768037 | n/a |
| *Suriana maritima* L. | TuTY1548 | MH767739 | MH768038 | n/a |
| *Corchorus aestuans* L. | LSC49 | MH767740 | MH768039 | MH768332 |
| *Corchorus aestuans* L. | TuTY2260 | MH767741 | MH768040 | MH768333 |
| *Triumfetta procumbens* G. Forst. | TuTY1462 | MH767742 | MH768041 | MH768334 |
| *Triumfetta procumbens* G. Forst. | TuTY1512 | MH767743 | n/a | MH768335 |
| *Trema orientalis* (L.) Blume | TuTY1528 | MH767744 | MH768042 | MH768336 |
| *Centella asiatica* (L.) Urb. | HXX18428 | MH767745 | MH768043 | MH768337 |
| *Centella asiatica* (L.) Urb. | TuTY1530 | MH767746 | MH768044 | MH768338 |
| *Pouzolzia zeylanica* (L.) Benn. | TuTY1431 | MH767747 | MH768045 | MH768339 |
| *Pouzolzia zeylanica* (L.) Benn. | ZLX12435 | MH767748 | MH768046 | MH768340 |
| *Lantana camara* L. | TuTY1461 | MH767749 | MH768047 | MH768341 |
| *Lantana camara* L. | TuTY2160 | MH767750 | MH768048 | MH768342 |
| *Phyla nodiflora* (L.) Greene | LSC39 | MH767751 | MH768049 | MH768343 |
| *Phyla nodiflora* (L.) Greene | TuTY2083 | MH767752 | MH768050 | MH768344 |
| *Premna serratifolia* L. | CHB18272 | MH767753 | MH768051 | n/a |
| *Premna serratifolia* L. | TuTY2683xs | MH767754 | MH768052 | n/a |
| *Stachytarpheta jamaicensis* (L.) Vahl | TuTY1437 | MH767755 | MH768053 | n/a |
| *Stachytarpheta jamaicensis* (L.) Vahl | TuTY2226 | MH767756 | MH768054 | MH768345 |
| *Volkameria inermis* L. | TuTY2076 | MH767757 | MH768055 | MH768346 |
| *Volkameria inermis* L. | TuTY2107 | MH767758 | MH768056 | MH768347 |
| *Tribulus cistoides* L. | TuTY1387 | MH767759 | MH768057 | MH768348 |
| *Tribulus cistoides* L. | TuTY1441 | MH767760 | MH768058 | MH768349 |
